# Prevalence and factors associated with rural mothers’ protection against tetanus: a cross-national analysis in 33 sub-Saharan African countries

**DOI:** 10.1093/inthealth/ihad103

**Published:** 2023-10-31

**Authors:** Wonder Agbemavi, Castro Ayebeng, Joshua Okyere, Emmanuella Acheampong, Vincent Bio Bediako

**Affiliations:** Department of Population and Health, University of Cape Coast, Cape Coast, Ghana; School of Demography, Australian National University, Canberra, Acton, ACT, Australia; Department of Population and Health, University of Cape Coast, Cape Coast, Ghana; Department of Population and Health, University of Cape Coast, Cape Coast, Ghana; Department of Nursing, College of Health Sciences, Kwame Nkrumah University of Science and Technology, Kumasi, Ghana; Department of Population and Health, University of Cape Coast, Cape Coast, Ghana; Department of Population and Health, University of Cape Coast, Cape Coast, Ghana; Ewim Polyclinic, Ghana Health Service, Cape Coast, Ghana

**Keywords:** rural mothers, sub-Saharan Africa, tetanus

## Abstract

**Background:**

Sub-Saharan Africa (SSA) and South Asia account for most new cases of tetanus. Despite efforts by the World Health Organization to eradicate tetanus, it still causes many maternal mortalities. We examined the prevalence and risk factors associated with tetanus protection among rural mothers in 33 SSA countries.

**Methods:**

Data were extracted from the most recent Demographic and Health Surveys of 33 SSA countries. A sample of 162 601 women from rural areas was drawn. Both descriptive and binary logistic regression analyses were conducted.

**Results:**

Overall, approximately half (49.3%) of rural mothers were protected against tetanus. The association between maternal age, education, marital status, working status, distance to the health facility and number of antenatal visits were statistically significant with rural mothers’ protection from tetanus. Also, relationship to the household head, household size and frequency of listening to radio, reading a newspaper and watching television were statistically significant in predicting rural mothers’ protection from tetanus.

**Conclusions:**

Policies and interventions by stakeholders must target high-risk populations, including adult women, those of poorer wealth status, those without media exposure and mothers with low educational attainment.

## Background

Tetanus is a bacterial infection caused by the *Clostridium tetani* bacterium.^1^ It is a toxin-mediated disease that is highly lethal, non-communicable and characterized by muscle spasms and autonomic nervous system dysfunction.^1^ Because of poor maternal tetanus toxoid immunization rates, maternal tetanus remains a public health issue worldwide.^2^

Worldwide, 75 million women and their newborns remain unprotected against tetanus,^3^ putting them at risk of contracting the disease, dying from it or suffering life-threatening health effects.^4^ Tetanus can develop in pregnant women and newborns due to poor hygiene following birthing.^2^ Maternal tetanus can develop during pregnancy or within 6 weeks of delivery.^4^ Tetanus in pregnancy is a problem for health equity since it disproportionately affects individuals who are poor, disadvantaged and without access to quality healthcare.^2^

Sub-Saharan Africa (SSA) and South Asia account for most new cases of tetanus.^3^ The two regions account for 82% of all tetanus cases globally.^5^ For instance, 77% of all deaths from tetanus occur in South Asia and SSA.^5^ Evidence from the World Health Organization (WHO) suggests the majority of SSA nations struggled to meet the tetanus immunization objective meant to be achieved in the region, which is responsible for about half of the world's maternal and neonatal tetanus fatalities.^1^ For instance, a study from Ethiopia reported that vaccine coverage may vary depending on the education and affluence of women.^6^ Additionally, it has been determined from the results of various studies that antenatal care (ANC) visits, women's education, income, distance to the health facility, place of residence, maternal age at first birth, women's employment status and media exposure are significantly linked to mothers who received tetanus vaccination.^6^

Despite efforts by the WHO to eradicate tetanus, it still causes a sizable number of maternal mortalities.^7^ Maternal tetanus causes at least 5% of maternal mortality in the world's poorest regions.^8^ Most of the obstacles to the eradication of maternal and neonatal tetanus are linked to socio-economic issues and healthcare systems.^8^ According to Gashaw,^6^ accelerating the elimination of maternal and neonatal tetanus in these nations, such as Mali, Nigeria, Sudan and Somalia, among others, will necessitate modifications to current maternal and neonatal tetanus elimination techniques, including innovations.^6^

Since many of the poorest and most neglected population groups are underserved regarding healthcare access, the tetanus problem is particularly severe in developing nations such as African countries.^9^ For instance, a previous study reported that mothers living in urban areas were more likely to receive the double tetanus vaccine than those in rural areas in Somalia and Sierra Leone.^10^ To the best of our knowledge, this is the first subregional analysis in SSA examining the associated factors of rural mothers’ protection from vaccination against maternal tetanus, despite the pervasiveness of tetanus in SSA.^1,3,5^ Although previous studies exist, these were done at the country level, and another in 10 East African countries. This situation presents a substantial knowledge gap, as the magnitude and dynamics of tetanus from the regional perspective are unclear. This study seeks to narrow the existing knowledge gap. The aim of our study was to examine the prevalence and risk factors associated with tetanus protection among rural mothers in 33 SSA countries. The findings of our study will expand the current literature on tetanus and demonstrate the general picture of what exists in the subregion (e.g. which country is doing better) and how public health measures can be put in place to solve the tetanus problem in specific countries and in the subregion as a whole.

## Methods

### Data source

**Figure 1.**
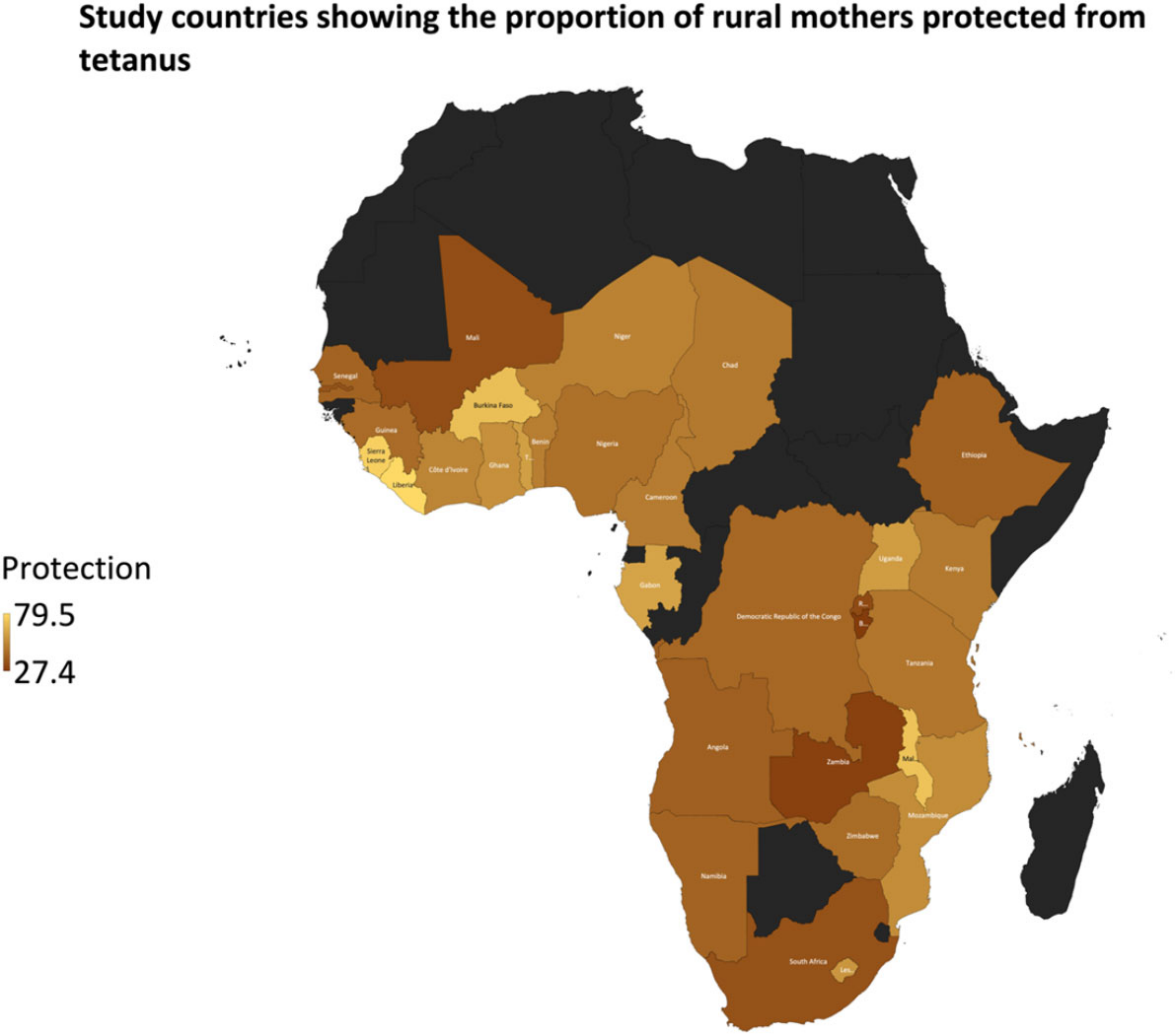
Map showing the proportion of mothers from rural areas protected against tetanus in 33 SSA countries. Dark shaded areas were not included in the study.

The analysis in this study is based on data extracted from the most recent Demographic and Health Surveys (DHS) (2010–2020) of 33 SSA countries that had the variables of interest included in the analysis (see Figure 1). The DHS is a nationally representative cross-sectional survey conducted every 5 y.^11^ The survey used a two-stage stratified cluster sampling approach to select samples of women in the reproductive age group (15–49 y) and men ages 15–64 y. The DHS is ideal for our study because it collects comprehensive information on various topics, including fertility, immunization coverage, tetanus toxoid injection and infant and maternal mortality. A sample of 162 601 women from rural areas was drawn from 33 SSA countries. MEASURE DHS approved the use of the dataset after reviewing our concept note. The dataset can be accessed at https://dhsprogram.com/methodology/survey/surveydisplay-491.cfm. We relied on the Strengthening the Reporting of Observational Studies in Epidemiology statement in conducting this study and writing the manuscript.^12^

### Study variables and measurements

#### Outcome variable

The outcome variable employed for this study was ‘protection against tetanus’. This was derived from the question on the number of tetanus toxoid injections received during the pregnancy of the most recent birth. The response was captured dichotomously, with ‘0’ for women who received less than two tetanus toxoid injections during the pregnancy of their most recent birth and ‘1’ for women who received two or more injections. This categorization is based on the WHO’s recommendation of at least two doses of tetanus toxoid injection during pregnancy, which is considered adequate.

#### Explanatory variables

From a review of the current literature,^2,13–15^ 10 explanatory variables were selected for the analysis. The explanatory variables include the women's age (15–19=1, 20–24=2, 25–29=3, 30–34=4, 35–39=5, 40–44=6, 45–49=7), level of education (no education=0, basic=1, secondary and above=2), wealth status (poorest=1, poorer=2, middle=3, richer=4, richest=5), marital status (never in union=0, married [including living with partner together]=1, formerly married [widowed, divorced and separated]=2), frequency of reading a newspaper or magazine (not at all=0, less than once a week=1, at least once a week=2), frequency of listening to the radio (not at all=0, less than once a week=1, at least once a week=2), frequency of watching television (not at all=0, less than once a week=1, at least once a week=2), working status (not working=0, working=1), distance to the health facility (big problem=0, not a big problem=1), number of ANC visits during the pregnancy of the most recent birth (less than four=0, four or more=1), ever had a terminated pregnancy (no=0, yes=1), relationship to the household head (head=0, wife=1, relative=2, not relative=3), household size, parity and country variable (Burkina Faso=0, Angola=1, Benin=2, …, Zimbabwe=32) (see Tables 1 and 2).

**Table 1.** Countries, survey years and percentage of rural mothers protected against tetanus in 33 SSA countries

				Mothers protected against tetanus	
Variable	Survey year(s)	Unweighted (n)	Weighted (n)	Not protected, n (%)	Protected, n (%)
Country				χ^2^=1.2e+04; p<0.001	
Burkina Faso	2010	7793	8694	2520 (28.99)	6174 (71.01)
Angola	2015	3671	3091	1872 (60.56)	1219 (39.44)
Benin	2017–2018	5124	5458	2761 (50.59)	2197 (49.41)
Burundi	2016–2017	7167	8340	6085 (72.96)	2255 (27.41)
Congo DR	2013–2014	7695	7661	4441 (57.97)	3220 (42.03)
Congo	2011–2012	4569	2193	815 (37.15)	1378 (62.85)
Cote d'Ivoire	2011–2012	6848	6346	3072 (48.41)	3274 (51.59)
Cameroon	2018	3373	3542	1818 (51.43)	1723 (48.66)
Chad	2014–2015	2840	3017	1584 (52.52)	1432 (47.48)
Ethiopia	2016	5568	6678	4025 (60.28)	2653 (39.72)
Gabon	2012	1454	521	198 (38.00)	323 (62.00)
Ghana	2014	2478	2278	1019 (44.72)	1259 (55.28)
Gambia	2019	3036	1816	1169 (64.37)	647 (35.63)
Guinea	2018	3780	3881	2200 (56.69)	1681 (43.31)
Kenya	2014	4617	4303	2244 (52.14)	2059 (47.86)
Comoros	2012	1023	1208	740 (61.29)	468 (38.71)
Liberia	2019–2020	2651	1758	361 (20.54)	1397 (79.46)
Lesotho	2014	1866	1832	781 (42.63)	1051 (57.37)
Mali	2018	9072	10 469	6984 (66.71)	3485 (33.29)
Malawi	2015–2016	11 034	11 893	3322 (27.94)	8570 (72.06)
Mozambique	2011	4853	5660	2593 (45.81)	3067 (54.19)
Nigeria	2018	13 902	13 543	7443 (54.96)	6100 (45.04)
Niger	2012	5737	6989	3458 (49.47)	3531 (50.53)
Namibia	2013	1587	1444	856 (59.30)	588 (40.70)
Rwanda	2019–2020	4834	5350	3621 (67.68)	1729 (32.32)
Sierra Leone	2019	4448	4304	1012 (23.53)	3291 (76.47)
Senegal	2019	2956	2519	1499 (59.48)	1020 (40.52)
Togo	2013–2014	6906	6195	2474 (39.94)	3721 (60.06)
Tanzania	2015–2016	5180	5101	2716 (53.24)	2385 (46.76)
Uganda	2016	8180	8039	3263 (40.59)	4776 (59.41)
South Africa	2016	599	473	309 (65.21)	165 (34.79)
Zambia	2018	4863	4587	3260 (71.08)	1326 (28.92)
Zimbabwe	2015	2897	3413	1927 (56.46)	1486 (43.54)
All countries		162 601	162 601	82 445 (50.70)	80 156 (49.30)

Source: Computed from the current DHS (2010–2020) of 33 SSA countries.

**Table 2.** Background characteristics of rural mothers protected against tetanus in SSA (N=162 601)

	Mothers protected against tetanus	
Characteristics	Not protected, n (%)	Protected, n (%)
Maternal age (χ^2^, p-value)	χ^2^=822.7512; p<0.001	
15–19	5913 (46.64)	6766 (53.36)
20–24	16 615 (45.65)	19 783 (54.35)
25–29	19 915 (50.15)	19 794 (49.85)
30–34	16 925 (52.50)	15 316 (47.50)
35–39	13 317 (54.98)	10 903 (45.02)
40–44	7215 (56.61)	5531 (43.39)
45–49	2544 (55.22)	2063 (44.78)
Education	χ^2^=1.8e+03; p<0.001	
No education	42 718 (55.29)	34 551 (44.71)
Primary	28 277 (48.74)	29 742 (51.26)
Secondary and above	11 449 (41.92)	15 863 (58.08)
Wealth index	χ^2^= 967.6026; p<0.001	
Poorest	27 155 (55.05)	22 172 (44.95)
Poorer	23 453 (51.36)	22 212 (48.64)
Middle	17 746 (48.27)	19 014 (51.73)
Richer	10 904 (46.84)	12 374 (53.16)
Richest	31 861 (42.10)	4383 (57.90)
Marital status	χ^2^=138.3660; p<0.001	
Never married	4027 (46.87)	4565 (53.13)
Married	73 266 (51.20)	69 846 (48.80)
Formerly married (separated/divorced/widowed)	5151 (47.28)	5745 (52.72)
Working status	χ^2^=380.2151; p<0.001	
Not working	30 328 (53.49)	26 370 (46.51)
Working	52 116 (49.21)	53 786 (50.79)
Distance to health facility	χ^2^=204.6568; p<0.001	
Big problem	40 282 (51.98)	37 210 (48.02)
Not a big problem	42 162 (49.54)	42 945 (50.46)
Number of ANC visits	X^2^=9.8e+03; p<0.001	
<4	52 388 (62.05)	32 035 (37.95)
≥4	30 057 (38.45)	48 120 (61.55)
Parity	X^2^=1.7e+03; p<0.001	
1	12 442 (41.68)	17 411 (58.32)
2	13 146 (47.88)	14 313 (52.12)
3	12 385 (50.30)	12 239 (49.70)
≥4	44 472 (55.13)	36 193 (44.87)
Ever had a terminated pregnancy	X^2^=0.0005; p=0.982	
No	71 087 (50.59)	69 434 (49.41)
Yes	11 357 (51.44)	10 722 (48.56)
Relationship to household head	X^2^=323.2049; p<0.001	
Head	10 118 (50.59)	9883 (49.41)
Wife	58 473 (51.87)	54 258 (48.13)
Relative	13 019 (46.74)	14 836 (53.26)
Not related	834 (41.45)	1179 (58.55)
Household size	χ^2^=525.7363; p<0.001	
1	169 (45.74)	200 (54.26)
2	1472 (43.69)	1898 (56.31)
3	7476 (43.82)	9587 (56.18)
4	11 084 (48.42)	11 808 (51.58)
≥5	62 244 (52.35)	56 663 (47.65)
Frequency of reading newspaper/magazine	χ^2^=311.1946; p<0.001	
Not at all	76 129 (51.41)	71 947 (48.59)
Less than once	4312 (44.55)	5367 (55.45)
At least once	1952 (41.44)	2759 (58.56)
Almost every day	52 (38.76)	82 (61.24)
Frequency of listening to radio	χ^2^=621.0698; p<0.001	
Not at all	42 396 (53.76)	36 467 (46.24)
Less than once	15 295 (48.03)	16 552 (51.97)
At least once	23 543 (47.84)	25 667 (52.16)
Almost every day	1211 (45.19)	1469 (54.81)
Frequency of watching television	χ^2^=238.4672; p<0.001	
Not at all	64 170 (51.52)	60 383 (48.48)
Less than once	9149 (48.70)	9639 (51.30)
At least once	8604 (47.85)	9379 (52.15)
Almost every day	521 (40.87)	755 (59.13)

Source: Computed from the current DHS (2010–2020) of 33 SSA countries.

Estimates are weighted.

### Statistical analysis

Two types of statistical analysis were performed: descriptive and binary logistic regression analyses. The descriptive analysis involved the bivariate analysis between the country variable and the outcome variable. It also showed the background characteristics numbers and percentages by the outcome variables. The associated χ^2^ test scores are also presented. Bivariate logistic regression was carried out to ascertain the statistically significant association between each selected explanatory variable and the outcome. Subsequently, multivariate logistic regression was employed to examine the significant association between the explanatory variables and the rural mothers’ protection against tetanus by adjusting for the net effects of all statistically significant variables. Using a 95% confidence interval (CI), the adjusted odds ratios (ORs) for each variable were determined. The data were handled and examined using Stata version 17 (StataCorp, College Station, TX, USA). The outcomes were sample weighted to address any under- or oversampling of participants from the total population. The survey's complex design was accounted for in the analysis by using the *svy* command in Stata in all the estimations. A multicollinearity test was conducted to rule out the presence of multicollinearity in the fitted multivariate logistic regression model.

## Results

Table 1 shows the prevalence distribution of rural mothers protected against tetanus by their country of residence in SSA. Overall, 49.3% of rural mothers are protected from tetanus in 33 SSA countries. Liberia has the highest proportion of rural mothers protected from tetanus (79.5%). This is followed by Sierra Leone (76.5%), Malawi (72.1%) and Burkina Faso (71.0%). Burundi recorded the least proportion (27.4%) of rural mothers who are protected from tetanus (see Figure 1). From the bivariate analysis, it can be observed that the prevalence of tetanus protection among rural mothers was statistically significantly different regarding the country of residence of mothers (see Table 1).

Table 2 shows the distribution of rural mothers’ protection against tetanus by their background characteristics in 33 SSA countries. The association between maternal age and mothers’ protection from tetanus was statistically significant (p<0.001). The prevalence of protection against tetanus among rural mothers is relatively high for the younger age categories compared with the older age categories. For instance, the highest uptake was among women 20–24 y of age (54.3%) and the lowest uptake of 43.4% was in rural mothers ages 40–44 y. When it comes to maternal education, the lowest uptake (44.7%) was recorded for mothers who had no education compared with those with primary education (51.2%) and secondary and above (58.1%). This association was statistically significant (p<0.001). Rural mothers with the poorest wealth status recorded a lower percentage (44.9%) of protection against tetanus compared with a higher percentage (57.9%) in the richest mothers. There was a statistically significant (p<0.001) difference in mothers’ protection against tetanus for those who were never married (53.1%), married (48.8%) and formerly married (52.7%). Mothers who indicated they were working had a higher percentage (50.8%) of tetanus protection compared with mothers who were not working (46.5%). This relationship was statistically significant (p<0.001).

Mothers who regard the distance to the facility as not being a problem had a higher percentage (50.5%) of protection against tetanus compared with those who considered the distance to be a big problem (48.0%). It was more common (61.5%) for mothers who had more than four ANC visits to be protected against tetanus than mothers who had less than four ANC visits (37.9%). A total of 58.3% of rural mothers who had a parity of one were protected from tetanus while 44.8% of those with a parity of four or more were protected from tetanus. This association was statistically significant (p<0.001). A total of 61.2% of rural mothers who read a newspaper/magazine were protected from tetanus compared with 48.6% of rural mothers who did not read a newspaper/magazine. Similarly, 54.8% of rural mothers who listened to radio almost every day were protected from tetanus compared with 46.2% of those who did not listen at all. Regarding the frequency of watching television, a higher proportion (59.1%) of mothers who watched television almost every day were protected from tetanus compared with those who did not watch television (48.5%).

### Factors associated with protection against tetanus

A binary logistic regression model was fitted to examine the factors statistically significantly associated with rural mothers’ protection against tetanus. Table 3 shows the unadjusted and adjusted ORs from the binary logistic regression and their respective CIs. The adjusted odds of protection compared with women ages 15–19 y was significantly less among older age group mothers, with protection decreased by 8% among mothers 25–29 y old (adjusted OR [aOR] 0.92 [95% CI 0.88 to 0.97]), 16% among those 30–34 y old (aOR 0.84 [95% CI 0.80 to 0.88]), 22% among those 35–39 y old (aOR 0.78 [95% CI 0.75 to 0.82]), 25% among those 40–44 y old (aOR 0.75 [95% CI 0.71 to 0.80]) and 22% among those 45–49 y old (aOR 0.78 [95% CI 0.72 to 0.84]). When it comes to rural mothers’ education status, mothers with primary education and secondary education and above were 1.24 (95% CI 1.21 to 1.28) and 1.45 (95% CI 1.40 to 1.51) times more likely to be protected from tetanus compared with mothers with no education. Again, the odds of protection from tetanus were higher for rural mothers who belonged to the poorer (aOR 1.08 [95% CI 1.05 to 1.12]), middle (aOR 1.14 [95% CI 1.10 to 1.17]), richer (aOR 1.11 [95% CI 1.07 to 1.15]) and richest (aOR 1.17 [95% CI 1.10 to 1.25]) categories than the reference category.

**Table 3.** Binary logistic regression results of factors associated with protection against tetanus

	Unadjusted estimates		Adjusted estimates	
Variable	OR	95% CI	AOR	95% CI
Maternal age				
15–19	Ref	Ref	Ref	Ref
20–24	1.05*	1.01 to 1.09	1.04	0.99 to 1.09
25–29	0.89***	0.85 to 0.92	0.92**	0.88 to 0.97
30–34	0.80***	0.77 to 0.84	0.84***	0.80 to 0.88
35–39	0.74***	0.71 to 0.78	0.78***	0.75 to 0.82
40–44	0.68***	0.65 to 0.72	0.75***	0.71 to 0.80
45–49	0.70***	0.65 to 0.74	0.78***	0.72 to 0.84
Country				
Burkina Faso	Ref	Ref	Ref	Ref
Angola	0.25***	0.23 to 0.27	0.19***	0.17 to 0.21
Benin	0.38***	0.35 to 0.41	0.29***	0.26 to 0.31
Burundi	0.15***	0.13 to 0.16	0.10***	0.09 to 0.11
Congo DR	0.27***	0.25 to 0.29	0.20***	0.19 to 0.22
Congo	0.66***	0.61 to 0.71	0.36***	0.33 to 0.39
Cote d'Ivoire	0.43***	0.40 to 0.46	0.40***	0.37 to 0.43
Cameroon	0.39***	0.36 to 0.43	0.25***	0.23 to 0.27
Ethiopia	0.24***	0.22 to 0.26	0.23***	0.21 to 0.25
Gabon	0.58***	0.52 to 0.66	0.36***	0.31 to 0.41
Ghana	0.46***	0.42 to 0.50	0.22***	0.20 to 0.24
Gambia	0.21***	0.19 to 0.23	0.10***	0.09 to 0.11
Guinea	0.30***	0.28 to 0.33	0.30***	0.27 to 0.32
Kenya	0.34***	0.31 to 0.36	0.21***	0.20 to 0.23
Comoros	0.29***	0.25 to 0.33	0.18***	0.15 to 0.21
Liberia	1.37***	1.23 to 1.52	0.71***	0.64 to 0.79
Lesotho	0.55***	0.49 to 0.61	0.25***	0.22 to 0.28
Mali	0.19***	0.18 to 0.20	0.15***	0.14 to 0.16
Malawi	0.98	0.91 to 1.03	0.66***	0.61 to 0.70
Mozambique	0.49***	0.45 to 0.53	0.34***	0.31 to 0.37
Nigeria	0.34***	0.32 to 0.36	0.24***	0.23 to 0.26
Niger	0.38***	0.35 to 0.41	0.37***	0.35 to 0.40
Namibia	0.28***	0.25 to 0.31	0.11***	0.10 to 0.13
Rwanda	0.19***	0.17 to 0.20	0.12***	0.11 to 0.13
Sierra Leone	1.25**	1.15 to 1.36	0.65***	0.59 to 0.71
Senegal	0.29***	0.26 to 0.32	0.20***	0.18 to 0.22
Chad	0.29***	0.27 to 0.32	0.30***	0.27 to 0.33
Togo	0.56***	0.53 to 0.60	0.44***	0.41 to 0.47
Tanzania	0.32***	0.30 to 034	0.21***	0.19 to 0.22
Uganda	0.59***	0.55 to 0.63	0.35***	0.32 to 0.37
South Africa	0.23***	0.19 to 0.27	0.08***	0.07 to 0.10
Zambia	0.16***	0.15 to 0.18	0.08***	0.07 to 0.09
Zimbabwe	0.31***	0.28 to 0.34	0.13***	0.12 to 0.15
Education				
No education	Ref	Ref	Ref	Ref
Primary	1.33***	1.30 to 1.36	1.24***	1.21 to 1.28
Secondary and above	1.75***	1.71 to 2.80	1.45***	1.40 to 1.51
Wealth index				
Poorest	Ref	Ref	Ref	Ref
Poorer	1.19***	1.16 to 1.22	1.08***	1.05 to 1.12
Middle	1.35***	1.31 to 1.39	1.14***	1.10 to 1.17
Richer	1.43***	1.38 to 1.47	1.11***	1.07 to 1.15
Richest	1.74***	1.65 to 1.83	1.17***	1.10 to 1.25
Marital status				
Never married	Ref	Ref	Ref	Ref
Married	0.82***	0.79 to 0.86	0.98	0.93 to 1.04
Formerly married (separated/divorced/widowed)	0.96	0.91 to 1.01	1.07	0.99 to 1.14
Working status				
Not working	Ref	Ref	Ref	Ref
Working	1.22***	1.20 to 1.25	1.12***	1.08 to 1.13
Distance to health facility				
Big problem	Ref	Ref	Ref	Ref
Not a big problem	1.15***	1.13 to 1.17	1.15***	1.12 to 1.17
Number of ANC visits				
<4	Ref	Ref	Ref	Ref
≥4	2.73***	2.67 to 2.78	2.82***	2.75 to 2.88
Ever had a terminated pregnancy				
No	Ref	Ref	Ref	Ref
Yes	1.00	0.97 to 1.03	0.99	0.96 to 1.02
Relationship to household head				
Head	Ref	Ref	Ref	Ref
Wife	0.97	0.94 to 1.00	0.97	0.93 to 1.00
Relative	1.19***	1.15 to 1.23	1.04	0.99 to 1.09
Not related	1.50***	1.37 to 1.63	0.09	0.99 to 1.20
Frequency of reading newspaper/magazine				
Not at all	Ref	Ref	Ref	Ref
Less than once	1.30***	1.24 to 1.35	1.01	0.96 to 1.06
At least once	1.46***	1.37 to 1.55	1.03	0.97 to 1.11
Almost every day	2.63**	1.24 to 2.15	1.21	0.90 to 1.63
Frequency of listening to radio				
Not at all	Ref	Ref	Ref	Ref
Less than once	1.27***	1.23 to 1.30	1.14***	1.10 to 1.17
At least once	1.27***	1.24 to 1.30	1.09***	1.06 to 1.12
Almost every day	1.43***	1.33 to 1.53	1.12**	1.03 to 1.21
Frequency of watching television				
Not at all	Ref	Ref	Ref	Ref
Less than once	1.14***	1.12 to 1.18	1.06**	1.03 to 1.10
At least once	1.19***	1.15 to 1.23	1.04*	1.00 to 1.09
Almost every day	1.54***	1.40 to 1.69	1.02	0.91 to 1.14

Source: Computed from the current DHS (2010–2020) of 33 SSA countries.

*p<0.05, **p<0.01, ***p<0.001.

Ref: reference category.

Rural mothers who reported working had increased odds of 1.12 (95% CI 1.08 to 1.13) of being protected against tetanus compared with mothers who were not working. Rural mothers who said distance to the health facility was not a big problem had increased odds of 1.15 (95% CI 1.12 to 1.17) of protection against tetanus than their counterparts who said distance to the health facility was a big problem. The number of ANC visits was statistically significant with rural mothers’ protection from tetanus. From Table 3, mothers who had four or more ANC visits were 2.82 (95% CI 2.75 to 2.88) times more likely to be protected from tetanus compared with mothers who had less than four ANC visits.

When it comes to the frequency of radio listening, rural mothers who listened to radio less than once (aOR 1.14 [95% CI 1.10 to 1.17]), at least once (aOR 1.09 [95% CI 1.06 to 1.12]) and almost every day (aOR 1.12 [95% CI 1.03 to 1.21]) had higher odds of protection from tetanus than mothers who did not listen to radio. Similarly, rural mothers who watched television less than once (aOR 1.06 [95% CI 1.03 to 1.10]) and at least once (aOR 1.04 [95% CI 1.00 to 1.09]) were more likely to be protected from tetanus than mothers who did not watch television. Regarding country of residence, rural mothers in Zambia had the lowest likelihood of 0.08 (95% CI 0.07 to 0.09) of being protected from tetanus compared with their counterparts residing in Burkina Faso.

## Discussion

This study examined the factors associated with tetanus protection among rural-dwelling mothers in SSA. Overall, less than half of mothers residing in rural areas in SSA were protected against tetanus. The scope of this study was wider than that of Belay et al.,^2^ as we looked at 33 countries as opposed to the 10 East African countries. Risk factors (age, wealth status, media exposure, a problem with distance to the health facility, marital status, frequency of ANC visits and maternal level of education) associated with tetanus protection among rural mothers are discussed in detail.

The study revealed that the odds of being protected against tetanus were significantly lower among adult women than adolescent girls. Similar findings were reported in a related study.^2^ However, the result contradicts the findings of a multicountry study^16^ that found that adults were more likely to be protected against tetanus than adolescent girls. Also, the finding contrasts with a related study from Nigeria^17^ that found no significant association between age and the likelihood of being protected against tetanus. A plausible explanation for this association could be that health education on vaccination against tetanus often targets adolescent girls and young women due to their likelihood of engaging in risky behaviours such as unprotected sex and having multiple sexual partners. Also, it is possible that adolescent girls might have received more recent and comprehensive tetanus vaccinations during their childhood or adolescence, ensuring higher levels of protection. In contrast, adult women might have received fewer booster doses of the vaccine, leading to reduced immunity against tetanus.

Consistent with previous studies,^2,16,18^ this study showed that higher maternal education was significantly associated with a higher likelihood of being protected against tetanus. This result can be explained from the perspective that higher maternal education predisposes or exposes women to health information and services that tend to increase their knowledge about the nature and importance of protecting themselves against tetanus, hence encouraging the uptake of preventive interventions such as the tetanus toxoid vaccine. Another possible explanation could be that mothers with a higher level of education are likely to be empowered to make autonomous decisions about their healthcare,^19,20^ including taking actions to protect themselves from tetanus.

Our findings show that being employed and having a higher wealth status corroborated related studies.^16,21,22^ One study by Yaya et al.^18^ found no significant association, but their sample size was smaller, which could explain the difference. Also, being employed empowers women to be autonomous in their healthcare decision making, which is likely to reflect in their uptake of preventive interventions. High wealth status increases the income and financial resources of women in rural areas to afford the cost of transportation, health screening and maternity costs that ordinarily would be a barrier to their likelihood of being protected against tetanus.^16^

In line with prior studies,^23,24^ this study showed that media (radio and television) exposure significantly increased the likelihood of women in rural areas being protected against tetanus. This observation is not surprising, as exposure to media increases women's access to information on tetanus and the need to take up preventive measures. Relatedly and surprisingly, the study revealed that a higher frequency of ANC visits increased the odds of women in rural areas being protected against tetanus. The result aligns with evidence from previous studies.^2,16^ During ANC sessions, healthcare providers offer education on a variety of potential health risks, including tetanus. Hence the more women attend ANC sessions, the more likely they are to be educated about the dangers of not taking preventive actions to protect themselves against tetanus.

Similar to the findings of Belay et al.,^2^ our study also found that women who did not have a problem with the distance to health facilities were 1.15 times more likely to be protected against tetanus compared with those who had a problem with distance, although this finding differs from Yeshaw et al.^16^ and Liyew and Ayalew.^25^ We think that a possible explanation for this could be related to transportation costs. Relatedly, the findings from this study are supported by prior literature^2,25^ that identified marital status as a significant factor associated with tetanus protection among rural-dwelling mothers in SSA.

## Policy implications

Our study highlights the need for advocacy and leveraging the available media platforms to advance awareness about tetanus prevention. Also, there is a need to expand pro-poor interventions such as health insurance schemes to ensure that women of low wealth status can access tetanus toxoid injections.

## Strengths and limitations of the study

The strength of this study lies in the use of a large sample size that is representative of 33 SSA countries. The implication is that the findings can be extrapolated to rural-dwelling women of reproductive age in SSA. Nevertheless, we cannot infer any causal pathways in the identified factors associated with rural mothers’ protection against tetanus in SSA due to the nature of the study design (cross-sectional). The questions on the number of tetanus toxoid injections received during the pregnancy were self-reported, hence there is a likelihood of recall bias. Also, important factors such as community norms and beliefs could not be assessed in this study due to the use of a secondary dataset. Future studies can explore the causal pathways in the factors associated with rural mothers’ protection against tetanus in SSA.

### Conclusions

In conclusion, the factors associated with rural mothers’ protection against tetanus in SSA include age, wealth status, media exposure, problem with distance to the health facility, marital status, frequency of ANC visits and maternal level of education. To further improve rural-dwelling women's protection against tetanus, policies and interventions must target high-risk populations, including adult women, those with a poorer wealth status, those without media exposure and those with low educational attainment. Given that the distance to the health facility was a significant factor, it is imperative to improve community-based health services to be able to reach women in rural areas who have a challenge with the distance to health facilities.

## Data Availability

The data are available online and can be found at https://dhsprogram.com.
